# Excellent response to erlotinib in breast carcinoma with rare EGFR mutation—a case report

**DOI:** 10.3332/ecancer.2020.1092

**Published:** 2020-08-25

**Authors:** Gunjesh Kumar Singh, Jyoti Bajpai, Shalaka Joshi, Kumar Prabhash, Anuradha Choughule, Asawari Patil, Sudeep Gupta, Rajendra Achyut Badwe

**Affiliations:** 1 Department of Medical Oncology, Tata Memorial Hospital, Mumbai 400012, India; 2 Department of Surgical Oncology, Tata Memorial Hospital, Mumbai 400012, India; 3 Department of Molecular Biology, Tata Memorial Hospital, Mumbai 400012, India; 4 Department of Pathology, Tata Memorial Hospital, Mumbai 400012, India

**Keywords:** triple negative breast carcinoma, metastasis, EGFR mutation

## Abstract

Triple negative breast carcinoma is a problematic subtype with poor outcomes. Many clinical trials are underway to find possible target to increase treatment options. Epidermal growth factor receptor (EGFR) has emerged as one such molecule which is over expressed in some of these patients and can be targeted by tyrosine kinase inhibitors. We describe a diagnostically challenging case of metastatic breast carcinoma, with extensive lung disease and poor Eastern Cooperative Oncology Group (ECOG) performance status, which expressed an uncommon EGFR mutation (Exon 21L861Q) and which benefitted from erlotinib following failure of all primary treatment modalities. The case uncovers the presence of these unusual mutations in breast carcinoma and highlights the importance of performing molecular analysis and the appropriate targeted therapy. This approach can be an important problem-solving tool, especially in cases where the patient is not fit for the other standard treatment options.

## Introduction

Breast cancer (BC) has become the most frequent malignancy in women worldwide as well as in India [1]. Considerable progress has been witnessed over years in the understanding of metastatic breast carcinoma (MBC), however, the overall 5-year survival rate still remains about 20% [2]. The triple negative breast carcinoma (TNBC) accounts for 10%–20% of all cases of BC and is the most challenging and difficult to treat subtype owing to the lack of standard prognostic and predictive markers, i.e., ER, PR and HER2 with an aggressive clinical course, and hence associated dismal prognosis [2]. Researchers are trying to explore the possibility of a definite target with this subtype which can be subjected to therapy. Several attempts and research done in this direction so far have discovered the potential role of EGFR in TNBC which is over expressed in a subset of these patients attributing to either gene amplification or activating mutations [3]. It is believed to have a major role in cell proliferation, survival, invasion and metastasis. Also, it is linked to lower hormone receptor levels and genomic instability [4]. This over expression is a hypothesis and perhaps can be evaluated for possible targeted therapy albeit, no such therapy is currently approved in this setting [3]. Herein, we report a treated case of MBC wherein this hypothesis was explored.

## Case presentation

A 64-year female, with a carcinoma of the left breast, underwent breast conservation surgery in 2002. Her histopathological examination (HPE) revealed intraductal carcinoma, grade 1 (pT1N0M0). The hormone receptor status was positive (ER+/PR+), while HER2 was negative. She further received loco-regional radiotherapy to the left breast. She was also given 5 years of hormone therapy with tamoxifen and was kept under regular follow up. In April 2019, she presented with rapidly progressing dyspnoea with poor Eastern Cooperative Oncology Group (ECOG) performance status (PS) 3.

**Figure 1.**
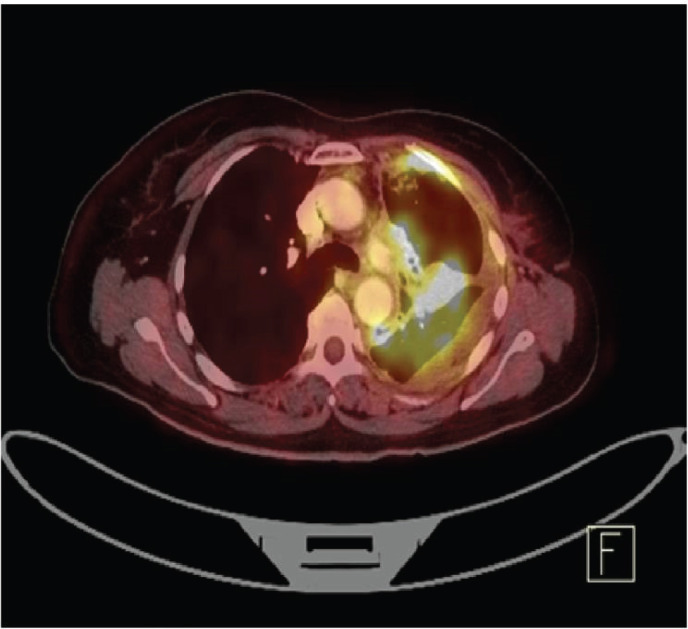
PET-CT showing metabolically active metastatic lesion in the upper lobe of left lung along with metabolically active moderate left side pleural effusion.

**Figure 2.**
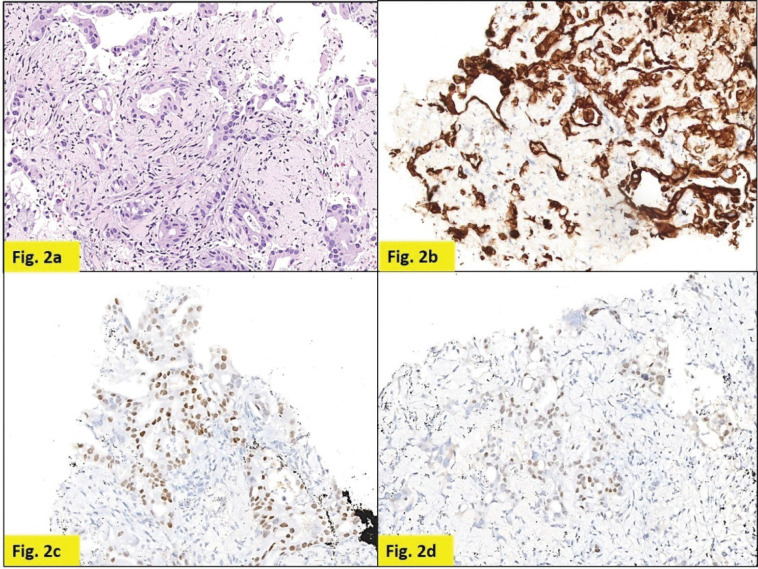
Pleural nodule biopsy (200×). (a) – H&E showing deposits of adenocarcinoma. (b) – Tumour cells expressing CK7. (c) – Tumour cells expressing CDX2. (d) – Tumour cells focally expressing GATA-3.

Staging positron emission tomography-computed tomography (PET-CT) was done which showed metabolically active lung disease (upper left lobe) and metabolically active nodular thickenings in the left mediastinal, diaphragmatic and coastal pleura along with metabolically active moderate left side pleural effusion (Figure 1). AN intercostal drainage tube was inserted and pleural fluid was sent for the analysis which showed presence of malignant cells. Pleural fluid cell block was tested with immunohistochemistry (IHC) to characterise the recurrence. They were positive for EMA, CK7 and negative for TTF1, GATA-3, CK20, PAX8, WT1 and Calretinin. However, occasional cells showed weak CDX2 positivity. CT guided pleural mass biopsy was performed which confirmed presence of adenocarcinoma. The tumour cells were diffusely positive for CK7, focally CDX2and GATA-3 while were negative for TTF-1, ER, PR, HER2 and CK20 (Figure 2a–d).

The pathological examination of both the pleural mass and the pleural fluid, favoured metastasis, with possible primary in the breast, gastrointestinal tract or lung. Normal clinical and radiological work up negated primary GI malignancy. Although negative IHC for TTF-1, calretinin and WT1 made primary lung and pleural involvement unlikely, a second primary in the lung could not be completely ruled out and was a remote possibility owing to the immunohistochemical pattern (ER negative, CK7+, CK20−) (Table 1) and long interval between the two malignancies. However, going by the patient’s previous history, positive IHC for CK7, EMA, GATA-3, negative IHC for TTF-1 and after extensive discussions and review, a final diagnosis of MBC was considered.

**Figure 3.**
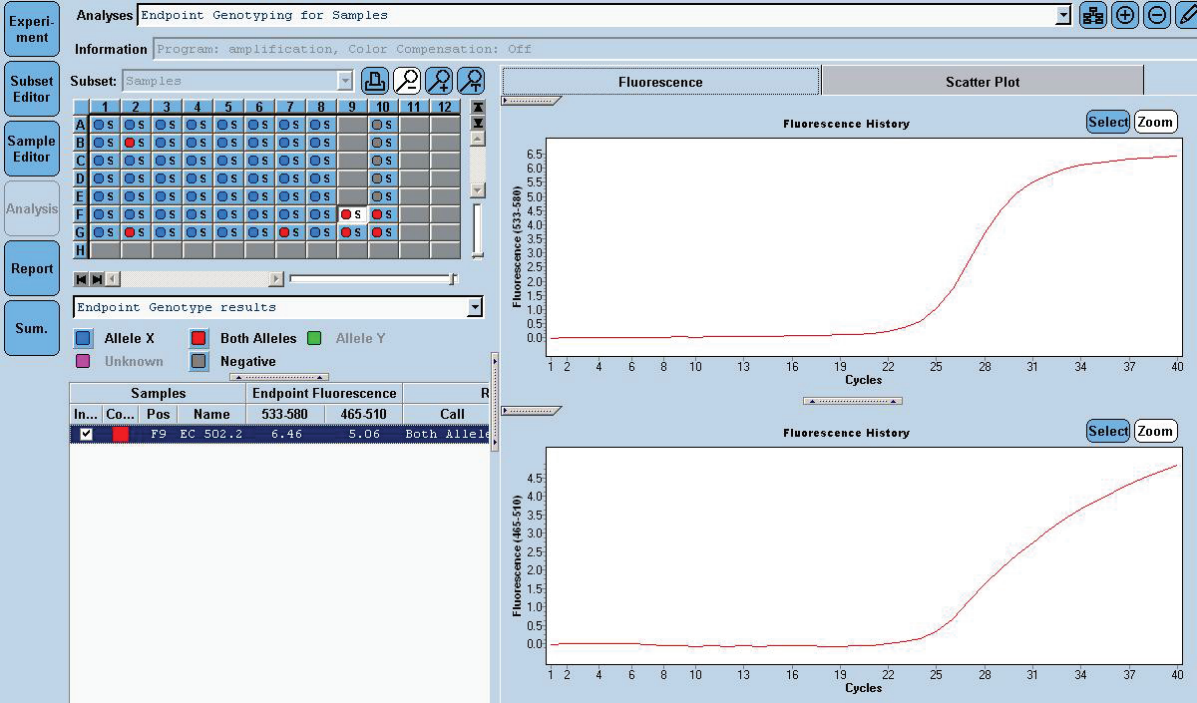
EGFR 21 L861Q Mutation was detected using home brew targeted TaqMan primer probes on the Real Time PCR platform LC 480 II.

**Figure 4.**
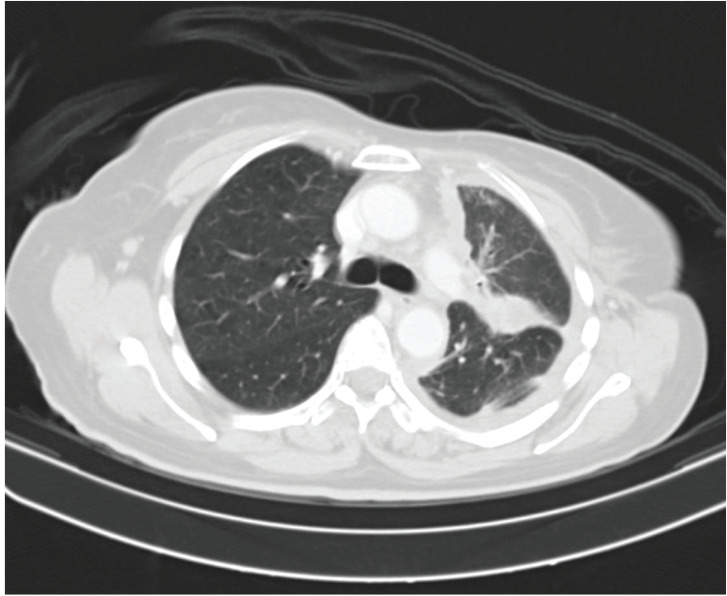
Contrast enhanced computed tomography (CECT) scan showing decrease in left sided pleural effusion with near stable diffuse pleural thickening along with decrease in consolidation.

She received one cycle of weekly paclitaxel 60 mg /m^2^ and carboplatin (AUC 1.5), however, the patient deteriorated with this standard chemotherapy. In view of poor PS and to have fair evidence to rule out primary lung carcinoma, reverse transcription polymerase chain reaction for EGFR was ordered which confirmed exon21 L861Q mutation positivity (Figure 3). The case was discussed in molecular tumour board and was started on erlotinib 150 mg daily dose on May 23, 2019 under close observation. She showed a quick response with symptomatic improvement in 2 weeks. The dyspnoea improved significantly and ECOG PS improved to 1. She was continued on the same drug and an assessment contrast enhanced computed tomography (CECT) scan was carried out on August, 14th, 2019 (after 3 months of starting the drug) which showed a partial response (Figure 4). She continued to be stable, with good general condition on erlotinib for 6 months when she progressed symptomatically as abdominal pain and dyspnoea on exertion. She developed rapidly progressive disease and succumbed to her illness on 20th December 2019.

## Discussion

EGFR is a receptor tyrosine kinase that belongs to the ErbB family. Activating mutations in EGFR gene leading to its over expression in breast cancer has been reported with a frequency of 13% to 78% [3]. however, has got geographic and ethnic variations (Americans – 0%–3.4%, Chinese 10% and 11.4%, and Koreans 1% to 2% of TNBCs) [5]. Tyrosine kinase inhibitors (TKIs) and anti-EGFR monoclonal antibodies are the current molecular target agents against EGFR available both as monotherapy and combination therapy. However, the use of EGFR inhibitors as an alternative treatment option for breast cancer has not been rewarding so far due to low clinical response rates. One reason for poor outcome is the activation of various alternative signalling pathways such as c-MET and IGF-1R which results in resistance to these targeted therapies [6]. According to Masuda *et al* [7], the disappointing results were related to the patient selection, which was not on the basis of EGFR expression. In a phase II trial, Baselga *et al* [8] evaluated the antitumor activity of gefitinib in advanced breast cancer and found a lack of clinical activity despite inhibition of EGFR phosphorylation. This could be because this type of breast cancer was independent of EGFR. They suggested future studies of gefitinib in combination with other agents and studies in selected subgroups of patients to identify the subsets of breast cancer patients. The index case presented years after primary treatment with recurrence/second primary in the form of pleural nodules and gross pleural effusion. HPE and IHC performed revealed simultaneous focal positivity of GATA-3 and CDX2 with a possibility of secondary deposits from either breast or GIT. However, there was no evidence of GIT involvement either clinically or radiographically. Other strong differential diagnosis based on the clinical presentation was second primary in the lung, especially because of long gap between the two malignancies. However, the negative IHC results for TTF-1 [9–11] favoured primary in the breast and after extensive discussion with pathologists and immunohistochemistry on multiple biopsies, the final decision was made to treat her like a metastatic breast carcinoma. Patient’s performance status deteriorated further with institution of standard second line chemotherapy, and she was nearly deemed unfit to receive any further chemotherapy. As a last resort, RTPCR for EGFR mutation analysis was carried out and it showed Exon 21 L861Q mutation positivity. The case could be narrowed down to two close differential diagnoses that is, either a metastatic breast carcinoma or a second primary in the lung as Exon 21 L861Q is also a well-known, but uncommon lung cancer mutation that confers sensitivity to TKIs [12]. Exon 21 L861Q mutation is known to activate the receptor tyrosine kinase and growth factor signalling pathway. This uncommon mutation is well known in adenocarcinoma lung and constitutes 2% of all EGFR mutations. Patients with this substitution mutation respond to TKIs (first generation – gefitinib, erlotinib, second generation – afatinib and third generation – osimertinib) [12]. Apart from lung cancer, Exon 21 L861Q mutation has also been reported in breast cancer, carcinoma urinary bladder and glioblastoma; however, its significance is largely unknown [13–15].

The case, however, was concluded as metastatic breast carcinoma. The fair expression of this target in this desperate situation convinced us to start the patient on erlotinib. Although this particular mutation in breast cancer has been reported fairly in literature, such welcoming response to TKI in this rare mutation is being reported for the first time [13]. Symptomatic improvement was evident soon after starting TKI and her ECOG-PS also improved considerably. She could have good quality of life for 6 months with this treatment. This case brought out the importance of ‘out of the box’ thinking. This simple test guided us towards the presence of a target and targeting the same could add six progression free months with improvement in the performance status and reasonable quality of life in this patient.

## Conclusions

Although rare, EGFR mutations have been reported in breast cancer, mostly seen in TNBC. Currently, this therapy still awaits approval for mass use in TNBC and hence routine EGFR testing is not advocated. However, it may still be opted in selected patients and an optimum response may be seen, especially when the standard chemotherapy options are exhausted or in cases of diagnostic dilemma with regard to metastasis with unknown primary.

## Conflicts of interest

None.

## Funding

None.

## Figures and Tables

**Table 1.** Incidence of GATA-3 and CDX-2 positivity in breast, GI and lung cancer.

IHC	Breast cancer	GI malignancies	Lung
GATA-3	80–90% [9]	<7% [9]	<10% [9]
CDX -2	Not seen	100% – Colorectal >50% – gastric adenocarcinoma >33% – pancreatobiliary tract carcinomas. [10]	40% – 50% of the non-mucinous adenocarcinomas (but along with TTF-1) [11]

GI – Gastrointestinal;

TTF-1 – Thyroid transcription factor-1.

## References

[1] Bray F, Ferlay J, Soerjomataram I (2018). Global cancer statistics 2018: GLOBOCAN estimates of incidence and mortality worldwide for 36 cancers in 185 countries. CA Cancer J Clin.

[2] Ro J, Cheng FT, Sriuranpong V (2016). Patient management with eribulin in metastatic breast cancer: a clinical practice guide. J Breast Cancer.

[3] Nakai K, Hung MC, Yamaguchi H (2016). A perspective on anti-EGFR therapies targeting triple-negative breast cancer. Am J Cancer Res.

[4] Changavi AA, Shashikala A, Ramji AS (2015). Epidermal growth factor receptor expression in triple negative and nontriple negative breast carcinomas. J Lab Physicians.

[5] Kim A, Jang MH, Lee SJ (2017). Mutations of the epidermal growth factor receptor gene in triple-negative breast cancer. J Breast Cancer.

[6] Buck E, Eyzaguirre A, Barr S (2007). Loss of homotypic cell adhesion by epithelial-mesenchymal transition or mutation limits sensitivity to epidermal growth factor receptor inhibition. Mol Cancer Ther.

[7] Masuda H, Zhang D, Bartholomeusz C (2012). Role of epidermal growth factor receptor in breast cancer. Breast Cancer Res Treat.

[8] Baselga J, Albanell J, Ruiz A (2005). Phase II and tumorpharmacodynamic study of gefitinib in patients with advanced breast cancer. J ClinOncol.

[9] Miettinen M, McCue PA, Sarlomo-Rikala M (2014). GATA3: a multispecific but potentially useful marker in surgical pathology: a systematic analysis of 2500 epithelial and nonepithelialtumors. Am J Surg Pathol.

[10] Silberg DG, Swain GP, Suh ER (2000). Cdx1 and Cdx2 expression during intestinal development. Gastroenterology.

[11] Kennedy MT, Jordan RC, Berean KW (2004). Expression pattern of CK7, CK20, CDX-2, and villin in intestinal-type sinonasaladenocarcinoma. J Clin Pathol.

[12] Zhang T, Wan B, Zhao Y (2019). Treatment of uncommon EGFR mutations in non-small cell lung cancer: new evidence and treatment. Transl Lung Cancer Res.

[13] Carey LA, Rugo HS, Marcom PK (2012). TBCRC 001: randomized phase II study of cetuximab in combination with carboplatin in stage IV triple-negative breast cancer. J Clin Oncol.

[14] Madison RW, Gupta SV, Elamin YY (2020). Urothelial cancer harbours EGFR and HER2 amplifications and exon 20 insertions. BJU Int.

[15] Xu H, Zong H, Ma C (2017). Epidermal growth factor receptor in glioblastoma. Oncol Lett.

